# 41.3 COULD CLOZAPINE REDUCE VIOLENT OFFENDING?

**DOI:** 10.1093/schbul/sby014.171

**Published:** 2018-04-01

**Authors:** Vishal Bhavsar, Kyriaki Kosidou, Nicola Orsini, Linnea Widman, James MacCabe, Christina Dalman

**Affiliations:** 1 Institute of Psychiatry, Psychology and Neuroscience, King’s College London; 2 Karolinska Institutet, Vishal Bhavsar, King’s College London, Institute of Psychiatry

## Abstract

**Background:**

Clozapine treatment may have beneficial effects on behavioural outcomes in psychotic disorders, including violent offending. Although clozapine and other antipsychotics have been linked to lower levels of violent behaviour, these have been primarily in small selected samples, and population-based estimates have been limited and imprecise.

**Methods:**

This study was a within-person cohort study based on linked prescription, hospitalization, and sociodemographic registers. We assessed the effect of clozapine treatment on the rate of violent and non-violent offending in the whole of Sweden, taking account of time-changing sociodemographic characteristics and the combination of violent and non-violent offences within individual convictions.

**Results:**

In a group of people treated with clozapine for psychotic disorders, violent offences were much less common during treatment than before. Effects on non-violent offences were smaller in magnitude, and lost precision on adjustments. There was a trend for the effects of antipsychotic treatment to increase with increasing age at initiation. Smaller but similar effects were observed for olanzapine. Clozapine rate reductions for violent offending were twice as strong for those with a history of alcohol-use disorders, compared to those without, RR for alcohol use disorders.

**Discussion:**

In patients with psychotic disorders, clozapine treatment is associated with a lower rate of violent offending compared to olanzapine. Clozapine might reduce offending through a direct effect on psychotic symptoms, or indirectly through changes in lifestyle, including use of alcohol.

